# A Biodegradable Copolyester, Poly(butylene succinate-*co*-ε-caprolactone), as a High Efficiency Matrix Former for Controlled Release of Drugs

**DOI:** 10.3390/pharmaceutics13071057

**Published:** 2021-07-10

**Authors:** Eduardo Galdón, Mónica Millán-Jiménez, Gloria Mora-Castaño, Antxon Martínez de Ilarduya, Isidoro Caraballo

**Affiliations:** 1 Department of Pharmacy and Pharmaceutical Technology, Faculty of Pharmacy, Universidad de Sevilla, 41012 Seville, Spain; egaldoncabrera@gmail.com (E.G.); momillan@us.es (M.M.-J.); gmora1@us.es (G.M.-C.); 2 Departament d’Enginyeria Química, Universitat Politècnica de Catalunya (ETSEIB), 08028 Barcelona, Spain; antxon.martinez.de.ilarduia@upc.edu

**Keywords:** poly(butylene succinate-*co*-ε-caprolactone), biodegradable polymer, ultrasound-assisted compression, hot melt extrusion, controlled release, nanostructured matrices

## Abstract

A biodegradable copolyester, poly(butylene succinate-*co*-ε-caprolactone) (PBS_CL), was used for first time as an excipient for pharmaceutical dosage forms using direct compression and hot processing techniques (ultrasound-assisted compression (USAC) and hot melt extrusion (HME)). Robust binary systems were achieved with hot processing techniques, allowing a controlled release of the drug. With only 12% *v*/*v* of PBS_CL, controlled release forms were obtained using USAC whereas in HME over 34% *v*/*v* of excipient is necessary. Amounts over 23% *v*/*v* allowed a long-extended release for more than 72 h following diffusional kinetic. Thanks to the high melting point of theophylline and the physicochemical properties of PBS_CL selected and synthesized, the structure of the excipient inside the USAC tablets and HME filaments corresponds to a continuum medium. A percolation threshold around 23% *v*/*v* was estimated, which agrees with a continuum percolation model. The polymer shows a high excipient efficiency value using HME and USAC. A nanostructured matrix with wall thicknesses lower than 0.1 µm was obtained. This leads to a very effective coating of the drug particles by the excipient, providing a slow and reproducible release. The present study therefore supports the use of PBS_CL, for the preparation of controlled release dosage forms using hot processing techniques.

## 1. Introduction

Advances in material design and engineering have led to the rapid development of new materials with increasing complexity and functions. Both degradable and non-degradable polymers have found wide applications in the field of controlled drug delivery [1,2,3,4]. As a result, the development of materials that are biocompatible and biodegradable and free from toxic metallic catalysts has gained great attention in recent years [5].

Aliphatic polyesters have received much attention since they are potentially biodegradable [6]. Moreover, these polymers have found applications in the biomedical field because they are biocompatible [7]. Within the aliphatic polyesters, poly(butylene succinate) (PBS) [8] and poly(ε-caprolactone) (PCL) [9] have emerged together with other more well-known polyesters such as poly(lactic acid) (PLA) or poly(glycolic acid) (PGA). PBS and PCL are semicrystalline polymers whose thermal properties (melting temperature (*T*_m_) or glass transition temperature (*T*_g_)) can be tuned by copolymerization. In fact, it has been found that poly(butylene succinate-*co*-ε-caprolactone) copolyester is crystalline for all compositions and crystallized isodimorphically for intermediate compositions [10]. 

Controlled release pharmaceutical forms provide a slow release rate of the drug, which becomes the rate limiting step in the arrival of the drug to the systemic circulation [11]. The reasons, among others, that have led to the formulation of controlled or sustained release drug delivery systems stem from the wish to decrease the number of daily administrations, improve compliance, and minimize side effects [12].

There are several methods to obtain the prolonged release of drugs. Direct compression remains the most widespread method to manufacture pharmaceutical dosage forms due to low cost of development as well as a high industrial production capacity [13]. 

In some cases, direct compression is not enough to obtain controlled release of the drug, with more complex methods being needed like hot processing techniques such as, for example, hot melt extrusion (HME) and ultrasound-assisted compression (USAC). 

These techniques have emerged as processing technologies for the development of controlled release solid pharmaceutical forms, for oral administration, and for implant preparation [14,15]. 

One of the limitations presented by these techniques is the fact that the polymers are exposed to high temperatures [16,17], requesting an adequate melting (*T*_m_) or glass transition temperature (*T*_g_). Additionally, the drugs should keep stability at the temperatures used. In the case of HME, the addition of plasticizer is sometimes necessary to increase the extrusion capacity of the polymer [18]. Even so, HME is a widely used technique in the manufacture of oral dosage forms [19].

USAC is a technology that combines the conventional compression process and ultrasound irradiation producing the heating, melting, and sintering of materials. Its application is less frequent than HME, but it can be useful in the preparation of formulations with solid dispersion to improve the bioavailability of poorly soluble drugs and formulation of sustained-release pharmaceutical forms, since it provides a better control of drug release with a smaller amount of excipient [20,21].

To explain the performance of this technique, the continuous percolation model [22,23] has been used, as well as the excipient efficiency (EE) and the slope of the Higuchi constant. EE provides a path to identify the ability of excipients to reduce the diffusion rate of a drug trapped in a network of this excipient [24].

In this study, drug delivery systems were prepared by direct compression, ultrasound-assisted compression and hot melt extrusion using, for the first time, the biodegradable copolyester, poly(butylene succinate-*co*-ε-caprolactone) with a selected feed composition (70/30 butylene succinate/ε-caprolactone) and theophylline as the model drug. This 70/30 composition of the copolyester was chosen because its thermal properties were found to be suitable for USAC and HME processing techniques. 

These systems were characterized from the physicochemical point of view. Furthermore, in vitro release studies were performed, in order to investigate the capacity of controlling the drug release of this new chemical entity processed with different technologies.

## 2. Materials and Methods 

### 2.1. Materials

**Figure 1:**
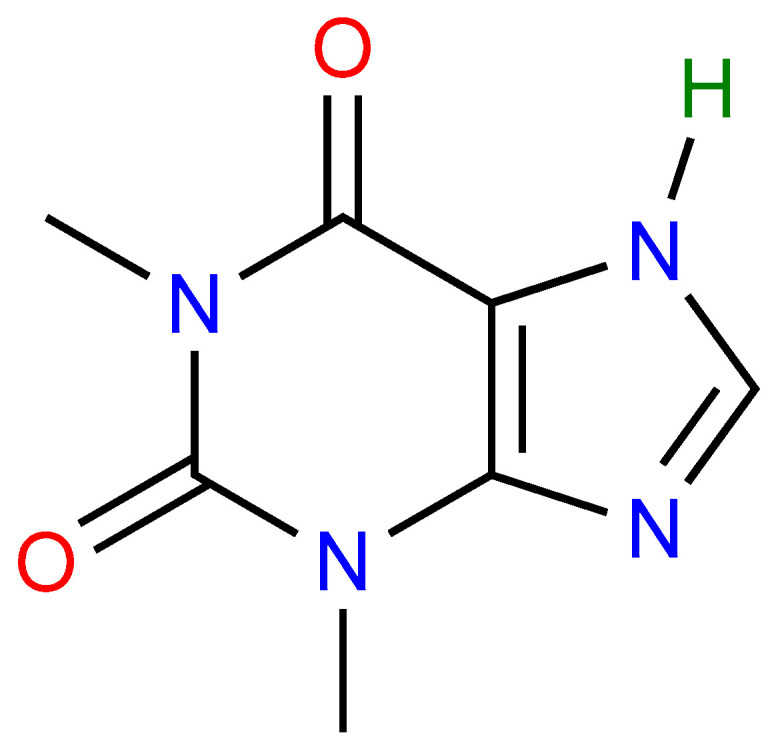
Structural formula of theophylline.

Anhydrous theophylline (Acofarma, Barcelona, Spain) was used as the model drug. Theophylline is a natural alkaloid derivative of xanthine (Figure 1) used as bronchodilator agent. It has experienced a renaissance due to preparations for oral use as a gradual release preparation [25]. It has been chosen as a model drug not only for its pharmacological characteristics but for its physicochemical and technological properties.

It is a drug with low solubility (7.36 mg/L at 25 °C in water) [26] and an intermediate partition coefficient (XLogP3 ≃ 0). As its pKa is 8.81, the main form in the gastrointestinal tract is the ionized form (Figure S1). From the point of view of pharmaceutical technology, it is a relatively complex drug due to the acicular shape of its crystalline form and its high capacity to charge electrostatically. 

Anhydrous theophylline was milled in a hammer mill and a fraction of drug particles size <500 µm was selected by sieving.

Dimethyl succinate (DMS), 1,4-butanediol (BD), titanium tetraisopropoxide (TTP) catalyst, and ε-caprolactone (CL) were purchased from Aldrich Co. All materials were used as received.

### 2.2. Methods 

#### 2.2.1. Synthesis and Characterization of Poly(butylene succinate-co-ε-caprolactone) 

Random copolyester with a feed molar ratio 70/30, butylene succinate/ε-caprolactone, (PBS_CL) has been synthesized by ring opening polymerization/polycondensation in the melt phase using the procedure previously reported [10]. The final polymer was taken from the reactor and used without further purification.

^1^H and ^13^C NMR spectra were recorded in a Bruker AMX-300 NMR instrument. The sample was dissolved in CDCl_3_ and spectra were referenced to the signal of the TMS internal reference. About 10 and 50 mg of sample were used for ^1^H and ^13^C NMR, respectively. Sixty-four scans were acquired for ^1^H and 5000–10,000 for ^13^C with 32-K and 64-K data points, respectively.

Molecular weight analysis was performed by GPC on a Waters equipment equipped with RI and UV detectors. A total of 100 μL of 0.1% (*w*/*v*) sample solution in 1,1,1,3,3,3-Hexafluoro-2-propanol was injected and chromatographed with a flow of 0.5 mL min^−1^. HR5E and HR2 Waters linear Styragel columns (7.8 mm × 300 mm, pore size 103–104 Å) packed with crosslinked polystyrene and protected with a precolumn were used. Molar mass average and distributions were calculated against PMMA standards.

The thermal behavior of PBS_CL copolyester was examined by differential scanning calorimetry (DSC), using a Perkin-Elmer DSC 8500. The thermograms were recorded from a 5 mg sample at heating and cooling rates of 10 °C·min^−1^ under a nitrogen flow of 20 mL·min^−1^. Indium and zinc were used as standards for temperature and enthalpy calibration. The glass transition temperature (*T*_g_) was taken as the inflection point of the heating DSC traces recorded at 20 °C·min^−1^ from melt-quenched sample, and the melting temperature (*T*_m_) was taken as the maximum of the endothermic peak appearing on the second heating trace. Thermogravimetric analysis was performed on a Mettler-Toledo TGA/DSC 1 Star System under a nitrogen flow of 20 mL·min^−1^ at a heating rate of 10 °C·min^−1^ and within a temperature range of 30 to 600 °C.

Flattened PBS_CL particles were frozen in liquid nitrogen and subsequently milled with a Restch ZM 200 equipment (Haan, Germany), using a 1.0 mm output sieve. Later they were sieved with a 500 µm sieve.

#### 2.2.2. Rheological Characterization of PBS_CL by SeDeM Method

Rheological studies of PBS_CL powder were carried out according to the SeDeM method developed by Suñé-Negre et al. [27]. This method unifies and standardizes several rheological parameters to provide quantitative information about the suitability of processing a powder through direct compression. Briefly, this expert system normalizes the rheological results from density, compressibility, flow, stability and lubricity tests leading to a result on a scale from 0 to 10. A value of 5 indicates that the product is acceptable for direct compression. 

The following rheological tests were performed: bulk density (ρbulk), tapped density (ρtapped), powder flow (t”), loss on drying (% LOD), interparticle porosity (IP), Carr’s Index (C), Hausner ratio (HR), cohesion index (CI), rest angle (α), percentage of particles measuring <45 μm (%*p* < 45), and homogeneity index (Iθ). The normalized results have been plotted in the SeDeM diagram [28]. The parametric index (PI), parametric profile index (PPI) and good compression index (GCI) were calculated based on the SeDeM approach to determine whether the PBS_CL is acceptable for direct compression.

#### 2.2.3. Preparation of Matrix Systems 

Blends of PBS_CL powder and theophylline at 12/88, 23/77, 34/66 and 47/53% *v*/*v* ratios have been mixed during 10 plus 10 min (Turbula mixer, Willy A. Bachofen, Basel, Switzerland). The optimum mixing time was calculated based on the drug content of five samples withdrawn at different time points. The drug content of the samples was determined by UV–Vis spectrophotometry (Agilent 8453, Agilent Technologies, Santa Clara, CA, USA) at 272 nm. Variation coefficient values lower than 5% were considered as appropriate.

Direct compression PBS_CL tablets were prepared in an eccentric tableting machine (Bonals A-300, Barcelona, Spain) using manual feeding and applying the maximum compression force accepted by the formulation. The tablets have a mean weight of 250 mg. They were prepared using 12 mm diameter flat punches with a dwell time of 0.18 s.

USAC tablets were prepared using an ultrasound-assisted tableting machine with a US generator coupled to the upper punch (Tecnea Engineering Srl, Bologna, Italy). An ultrasonic energy of 650 J was applied to the powder during the compaction process at a frequency of 20 kHz. The tableting machine was equipped with flat cylindrical punches of 11 mm. The parameters established for the proper compression were compression pressure 0.3 MPa, compaction time 6 s, cool time 9 s, and detach time 0.5 s. USAC tablets were prepared with 250 mg of formulation.

Hot melt extrusion filaments were prepared in order to test the suitability of the polymer to be processed with this technological method. The polymer and the anhydrous theophylline were studied in different proportions (34/67 and 45/55% *v*/*v*). The physical mixtures were extruded using a co-rotating twin screw extruder (Haake MiniLab, Thermo Electron, Karlsruhe, Germany), operating at a screw speed of 50 rpm and the extrusion temperature was set to 125 °C for both formulations. Uniform strands of extrudate were collected during steady state extrusion only for the 45/55 ratio. On the other hand, the strand with the proportions 34/67 did not have adequate characteristics to be used.

#### 2.2.4. Matrix Systems Characterization

The tablets and filaments obtained were characterized as follows:

Physical dimensions. The tablet average weight and the standard deviation (SD) were obtained from six individually weighed tablets (Sartorius CP224S, Gottingen, Germany). The external dimensions (height and diameter) were determined as the mean of six tablets from each batch using the digital micrometer (VWR International, Leuven, Belgium). The multidimensional image analysis software Nis-elements BR (Nikon Corporation, Chiyoda-ku, Tokyo, Japan) was used to calculate the lateral surface area of the filaments. 

Drug release assays were carried out in a USP apparatus 2 Sotax AT7 Smart, (Sotax, Allschwil, Switzerland) using 900 mL of deaerated water maintained at 37 ± 0.5 °C, during 24 h at 50 rpm. The percentage of drug released was measured with an UV–vis spectrophotometer Agilent 8453 (Agilent, CA, USA) at a wavelength of 272 nm. The test was performed in triplicate for each batch. Sink conditions were met throughout the dissolution test.

Kinetic adjustments. Drug release data (M_t_/M_∞_ ≤ 0.6) were analyzed according to zero order, Higuchi [29], Korsmeyer et al. [30], and Peppas and Sahlin [31]. Equations (1)–(4):

(1) $$\frac{M_{t}}{M_{\infty }}={\;k}_{0}t$$

(2) $$\frac{M_{t}}{M_{\infty }}={\;\mathrm{kt}}^{1/2}$$

(3) $$\frac{M_{t}}{M_{\infty }}=k_{k\;}t^{n}$$

(4) $$\frac{M_{t}}{M_{\infty }}=k_{d}t^{m}+k_{r}t^{2m}$$

where M_t_/M_∞_ is the fraction of drug released at time t (the drug loading was considered as M_∞_). k is the Higuchi’s release rate constant. k_k_ is the Korsmeyer’s kinetic constant, t the release time, n the release exponent that depends on the release mechanism and the shape of the matrix tested [32], k_d_ and k_r_ are, respectively, the diffusion and relaxation rate constants, and finally m, which is the purely Fickian diffusion exponent for a device of any geometrical shape that exhibits controlled release.

Estimation of the excipient efficiency. The excipient efficiency was calculated for the different batches by applying the following Equation (5) according to Caraballo [33]:

(5) $$\mathrm{EE}=\frac{\varepsilon }{b}$$

where ε is the total porosity of the matrices, b is the Higuchi’s release rate constant. The total porosity is calculated with the known values of volume and weight of tablets according to the following Equation (6):

(6) $$\in =\frac{\mathrm{Vreal}-\mathrm{Vtheor}}{\mathrm{Vreal}}\times 100$$

where Vreal is the real volume of the tablets and Vtheor the theoretical volume of the tablets, obtained as the PBS_CL volume calculated from its true density obtained by Helium picnometry, Pentapycnometer 5200E (Quantachrome Instruments, Boynton, FL, USA). Drug volume was not taken into account due to its soluble behavior.

Scanning electron microscopy. The surface of samples, tablets obtained by USAC, and DC and HME filaments were evaluated at the Microscopy Service of the CITIUS in the University of Seville by using scanning electron microscopy (SEM) with a FEI TENEO electronic microscope (FEI Company, Hillsboro, OR, USA), operating at 5 kV. Tablets were previously coated with a 10 nm thin Pt layer with Leica EM SCD500 high vacuum sputter coater.

## 3. Results and Discussion 

### 3.1. PBS_CL Characterization

**Scheme 1:**
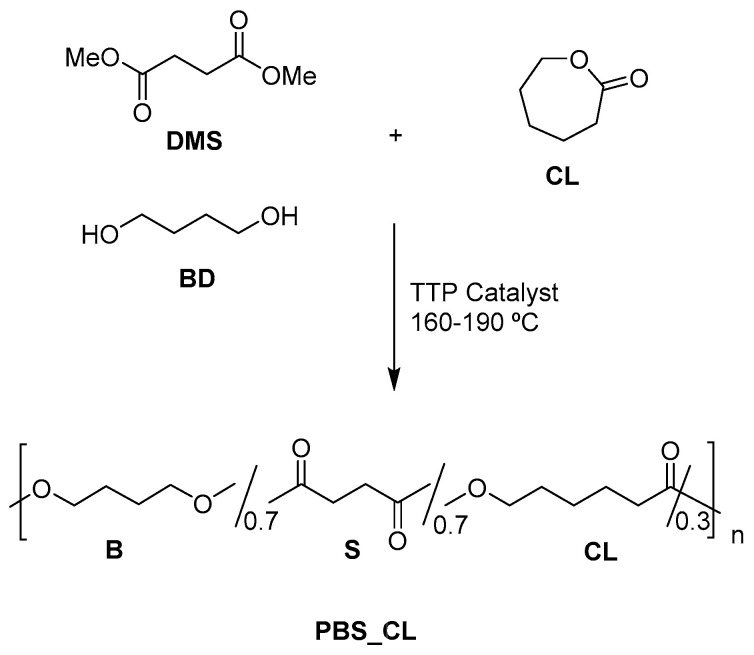
Synthesis route to PBS_CL copolyester.

**Figure 2:**
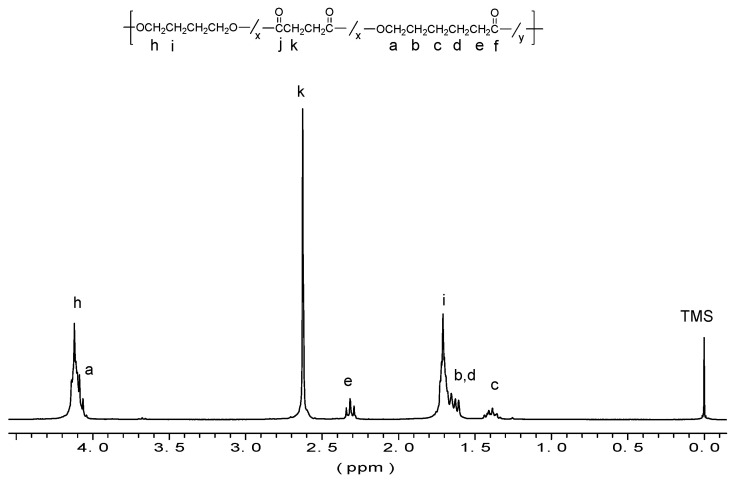
^1^H NMR spectrum of PBS_CL copolyester with peak assignments.

PBS_CL copolyester with a BS/CL feed molar 70/30 was synthesized using the synthetic method reported by Safari et al. [10] (Scheme 1). This composition of the copolyester was chosen because its thermal properties could be suitable for USAC and HME processing techniques (solid polymer at room temperature with melting point around 90 °C and a crystallization temperature around 35 °C in order to obtain a rapid recrystallization of the filament, once extruded). A polymer with high molecular weight was obtained (*M*_n_= 24.300 g·mol^−1^ and *Ð* = 2.1). The infrared spectrum of PBS_CL copolyester is depicted in Figure S2. It shows the characteristic bands associated with the carbonyl ester groups as a broad peak centered at 1714 cm^−1^ and C-O stretching absorption at 1160 cm^−1^. Other absorption bands corresponding to the stretching and bending of methylene groups are observed at 2949 and 1421 cm^−1^, respectively. Moreover, Figure 2 shows the ^1^H NMR spectrum with peak assignments. The composition of the copolyester was calculated by integration of the signals corresponding to methylene next to the carbonyl of the caprolactone unit (e) and the methylene signals of the succinate unit (k). It was observed that the calculated composition was very near to feed composition (73/27). On the other hand ^13^C NMR was used to determine the microstructure of the copolymer. Some signals of the spectra split into three or four peaks due to sequence distributions. By deconvolution of these peaks, the degree of randomness could be determined. It was very close to 1, proving that the copolyester had a random microstructure (Figure S3).

**Figure 3:**
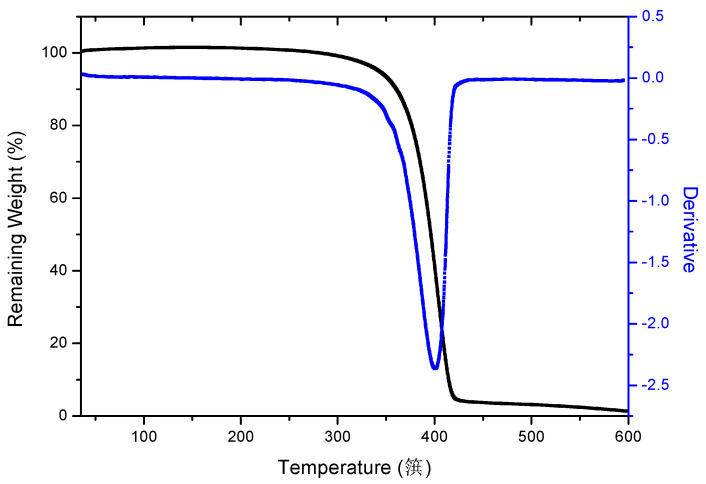
TGA thermograms of PBS_CL copolyester recorded under nitrogen atmosphere.

It was thermally stable up to 250–300 °C as observed by TGA analysis (Figure 3).

This thermal stability was high enough for ensuring the used processing techniques without deterioration of properties. On the other hand, the copolyester was observed to be semicrystalline with a melting temperature and enthalpy of 90.2 °C and 51.5 J·g^−1^, respectively. Full data of PBS_CL characterization are reported in Supplementary Materials.

The hydrolytic/enzymatic degradation of PBS_CL results in 1,4-butanediol, hexanoic acid, and butanedioic acid that are biocompatible and non-toxic [34,35,36].

### 3.2. Matrix Systems Characterization

Tablets of PBS_CL and theophylline were successfully obtained by USAC and DC for all the prepared blends. 

However, HME filaments were only obtained for PBS_CL contents higher than 34% *v*/*v*. This is in agreement with the results previously obtained by Verstraete et al. for different polymers [32,33] and with past unpublished research by our group. Furthermore, Viidik et al. [34], using polycaprolactone and theophylline blends, reported difficulties obtaining filaments by HME at 60 to 80% *w*/*w* of drug without the use of a plasticizer.

The physical characterization of the different batches is summarized in Table 1. The differences in physical dimensions are a consequence of the methods used during the preparation. Weights were maintained constant for all systems.

Results are expressed in *v*/*v*% to facilitate the interpretation of the behavior of the systems, especially on the basis of excipient efficiency and percolation theory approaches.

### 3.3. Rheological Results According to SeDeM Method

Rheological parameters of PBS_CL were obtained according to the described methodology. This preformulation tool has been chosen as it provides very useful information about the suitability of substances to be directly compressed. Moreover, this method provides information about industrial processing of powdery substances.

**Figure 4:**
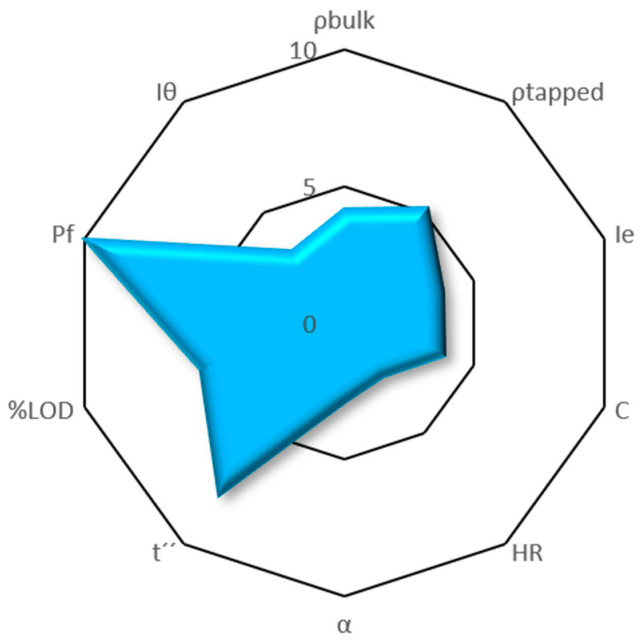
SeDeM diagram of PBS_CL.

Table 2 shows the obtained results of the studies with normalized values as well as the mean of each incidence, and Figure 4 shows the SeDeM diagram where these rheological results have been plotted.

PBS_CL shows moderate characteristics for direct compression in all the studied properties. The SeDeM Expert System proposes three indexes to determine whether a powder is acceptable for direct compression: parametric index (PI), parametric profile index (PPI) and good compression index (GCI). Values higher than 0.5, 5, and 5, respectively, indicate that the substance can be processed through direct compression [27,37]. The three indexes were calculated according to this method. The good compressibility index (IGC) of PBS_CL, 5.29, is similar to other excipients used in direct compression [38] and overcame the acceptable value. PI and PPI results were very close to the acceptable values (PI: 0.4; PPI: 4.89). The obtained results confirm that the polymer has adequate compression properties, even in the range of direct compression tableting excipients.

### 3.4. Drug Release Results

**Figure 5:**
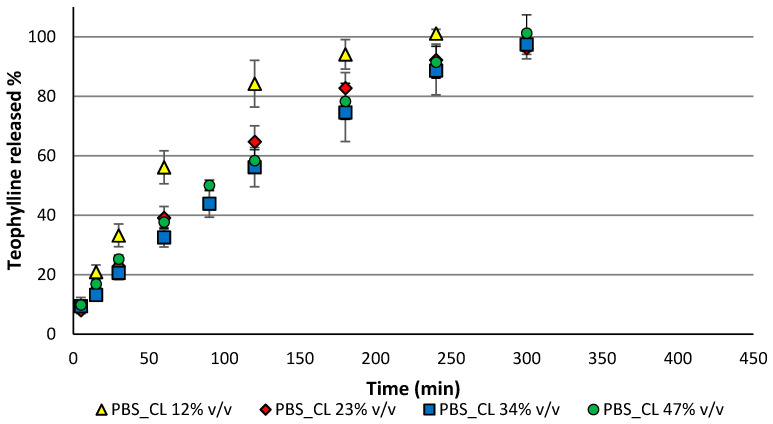
Dissolution profiles for PBS_CL tablets obtained by direct compression.

Drug dissolution studies from tablets obtained by the direct compression method with PBS_CL are shown in Figure 5. A rapid dissolution profile can be observed for tablets with 12% *v*/*v* of PBS_CL (less than 240 min for 100% released). The release profiles of tablets with higher amounts of PBS_CL (23, 34 and 47% *v*/*v*) were very similar between them and showed a higher capacity for the prolonged release of the drug (300 min for 100% theophylline released). The integrity of the tablets was lost in all cases during the dissolution test. 

**Figure 6:**
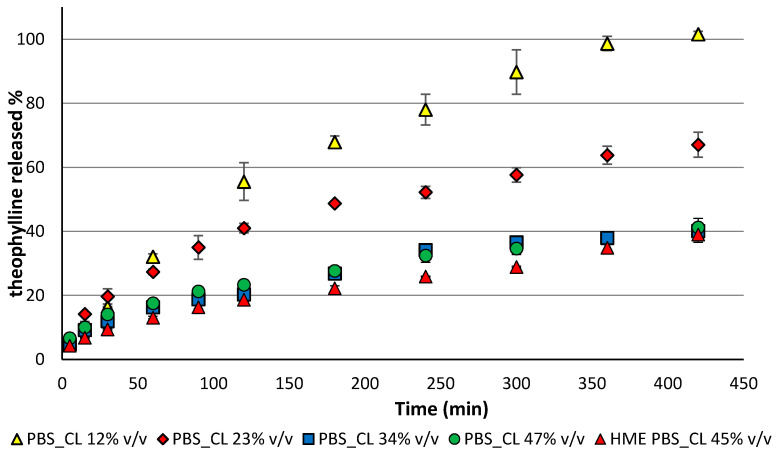
Dissolution profiles for PBS_CL tablets obtained by ultrasound-assisted compression and HME filaments.

Drug dissolution studies from tablets obtained by the ultrasound-assisted compression method with PBS_CL are shown in Figure 6. It A prolonged release dissolution profile can be observed for tablets even with only 12% *v*/*v* of PBS_CL (360 min for 100% released). The integrity of these tablets was also lost during the dissolution test. When the amount of PBS_CL increases to 23% *v*/*v*, the release profile of theophylline drops significantly, releasing only 67% of the drug after 420 min. The drug release drops again significantly for concentrations above 23% *v*/*v*, releasing only 42% of the drug in 420 min while keeping their shape and dimensions after the dissolution test. 

For concentrations higher than 23% *v*/*v* of excipient, an incomplete drug release is observed and a coherent skeleton of the insoluble excipient appears that provides integrity to the tablet. This indicates the existence of an excipient infinite cluster able to develop mechanical resistance to the disintegration, controlling the release of the drug.

This result is in agreement with the critical point previously reported by Caraballo et al. based on a continuum percolation model. The excipient percolation threshold was estimated to be in the range of 13.4 to 20.2% *v*/*v* of polymer (Eudragit RS) for systems compacted by USAC [22].

In a conventional sustained release tablet, the limited accessibility of many drug particles to the dissolution medium can be assumed since they are encapsulated by polymeric materials [39]. This effect is clearly increased when the excipient particles undergo a sintering process and are transformed in a quasi-continuum medium. In this case, the coating of the drug is much more effective.

In order to perform a better characterization of the obtained drug delivery systems, the excipient efficiency has been calculated for all of them. The excipient efficiency is a parameter developed to quantify the ability of an excipient to control the drug release [40]. As can be observed in Table 3, PBS_CL USAC tablets show a higher EE value than PBS_CL direct compression tablets. This is attributed to the sintering process that particles usually undergo with the USAC technique [28].

**Figure 7:**
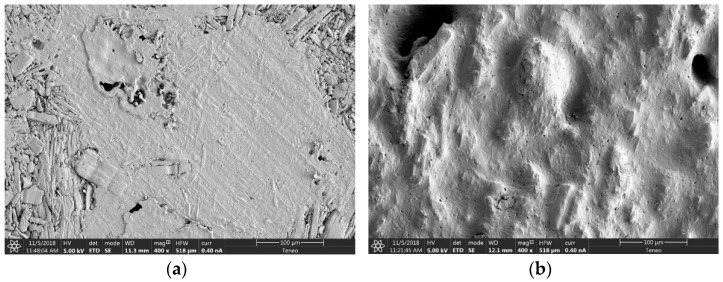
SEM images, magnification 400×, of PBS_CL tablets obtained by USAC (47/53% *v*/*v*) (**a**) and filaments obtained by HME (45/55% *v*/*v*) (**b**) before release of drug.

Hot melt extrusion filaments were prepared to study the suitability of the PBS_CL for the HME method. The release profile corresponding to 45% *v*/*v* is shown in Figure 6. The obtained filaments show a great ability to control drug release despite their thin shape. This is reflected by the high excipient efficiency obtained for systems prepared by hot melt extrusion (see Table 3). They released only 40% of theophylline in 480 min while maintaining their shape and dimensions. Compared to PBS_CL USAC systems with similar polymer contents (see HME PBS_CL 45% *v*/*v* versus USAC PBS_CL 47% *v*/*v* in Figure 6), the filaments obtained by HME show a slightly lower release rate in the first 30 min. This can be attributed to a better coating of the drug particles situated on the outer surface of the system, as can be clearly observed in Figure 7.

These results are concordant with those previously reported by Galdón et al. [28]. 

### 3.5. Kinetic Analysis

Regarding kinetic studies, drug release profiles from PBS_CL DC tablets for concentrations above 34% *v*/*v* seem to fit the Zero order model, according to the kinetic parameters shown in Table 3. The significant difference between k_0_ values reflects the rapid dissolution rate of tablets with 12% *v*/*v* of PBS_CL compared with tablets with higher concentrations. Moreover, kr values in the Peppas and Sahlin models show a certain contribution of the erosion mechanism. This agrees with the loss of integrity of these tablets during the dissolution test.

According to Peppas and Sahlin kd and kr values for HME filaments and USAC tablets with concentrations above 12% *v*/*v* PBS_CL, diffusion seems to be the main release mechanism whereas relaxation-erosion mechanism is almost non-existent (see Table 3). This is in agreement with the fact that these tablets behave as inert matrices, keeping their integrity during drug dissolution. 

The *n* value in the Korsmeyer model indicates which is the main release mechanism depending on the geometric shape of the systems, based on the aspect ratio parameter [30]. As the aspect ratio value of our system ranges from 5 to 9, *n* values around 0.45 (see Table 3), this confirms that diffusion is the main release mechanism of our systems.

Therefore, both release kinetics studies demonstrate that polymer hydrolysis does not significantly influence the release kinetics nor the tablet integrity. Therefore, the release of the model drug is driven by a diffusion-controlled mechanism.

On the other hand, USAC tablets with a PBS_CL concentration of 23% *v*/*v* do not fit any kinetic model, suggesting that this concentration of PBS_CL is very close to a critical point, i.e., a geometrical phase transition causing a high variability and erratic release profiles.

### 3.6. Internal Structure Study

To explain the release behaviour of the obtained systems, the structure of the matrices was studied by SEM.

**Figure 8:**
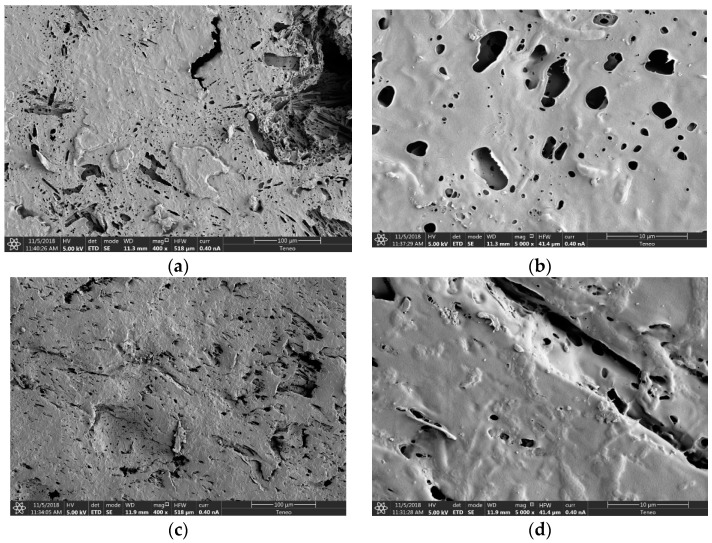
SEM images of PBS_CL tablets obtained by USAC (47/53% *v*/*v*) and filament obtained by HME (45/55% *v*/*v*) after drug release. (**a**,**b**) USAC tablets at 400× and 5000×; (**c**,**d**) HME filament at 400× and 5000×.

Due to the sintering of the polymer in USAC and the fusion process in HME, a very tight scaffold is formed (Figure 8).

**Figure 9:**
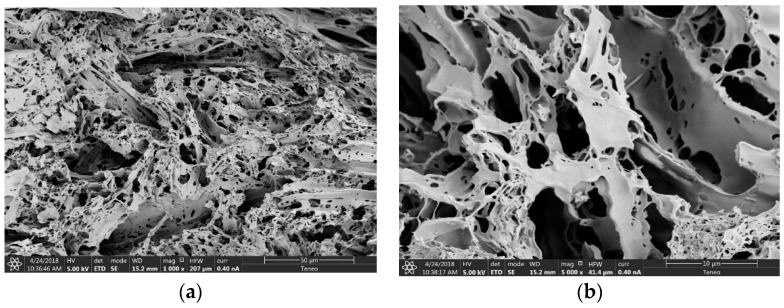
SEM images of PBS_CL tablets obtained by USAC (23:77% *v*/*v*) after drug release. Detail of the nanostructured matrix (**a**) 1000× (**b**) 5000×.

In order to reveal the inner structure of the matrix, we studied tablets obtained by USAC at 23:77% *v*/*v* after the drug was released for 72 h (Figure 9). It has been found that the excipient has a great efficacy coating the drug with layers less than 0.1 µm thick, forming what we have called a nanostructured matrix system.

Previous studies have used microporous films and nanostructured matrices prepared by thermally induced phase separation. Unfortunately, most biocompatible materials useful for the preparation of such microporous films are sub-optimal for drug delivery due to poor processability and/or drug encapsulation properties [41,42,43]. In the systems studied in the present paper, the drug is incorporated into the polymer before the preparation of the nanostructured matrix and, thanks to the properties of PBS_CL, the obtained nanostructure matrices can load a high amount of drug, controlling its release rate without a burst effect.

## 4. Conclusions

The results of this study demonstrate the suitability of the biodegradable polymer PBS_CL for preparing sustained release systems in proportions as low as 12% by volume (10% by weight) of excipient, using hot processing techniques (USAC and HME). 

The high excipient efficiency showed by the polymer using USAC and HME derives from the formation of a nanostructured matrix capable of containing high drug loads. This structure also results in a very high ability to control drug release. On the basis of percolation theory, this is due to a decrease in the critical point of the excipient from 30–35% *v*/*v*, which corresponds to a particulate system, to a range of 13.4 to 20.2% *v*/*v* of polymer, corresponding to a continuum percolation model.

The behavior of the nanostructured matrices introduced in the present work has been explained based on the study of the release kinetics, the excipient efficiency, the direct observation of their structures before and after drug leaching, and the percolation thresholds of the systems. This may be of help in the estimation of the design space for these new formulations, in line with the ICH Q8 Guidelines and the quality by design concept [44]. 

On the other hand, PBS_CL would also be adequate for the preparation of other kinds of drug delivery systems for the controlled release of drugs, e.g., implants, 3D printed dosage forms, etc.

## Figures and Tables

**Table 1:** Dimensions of PBS_CL tablets obtained by DC and USAC and HME filaments.

Batch	PBS_CL Content (%vol)	Theophylline Content (%vol)	Total Weight (mg)	Diameter (mm)	Thickness (mm)	Volume (mm^3^)	Initial Porosity (ε_0_)
12:88 DC	12	88	246.1 ± 4.9	12.10 ± 0.01	2.001 ± 0.244	218.79	23.37
23:77 DC	23	77	242.6 ± 2.1	12.13 ± 0.08	1.869 ± 0.224	205.26	17.77
34:66 DC	34	66	240.4 ± 0.8	12.25 ± 0.23	1.764 ± 0.010	204.57	16.58
47:53 DC	47	53	238.9 ± 1.6	12.14 ± 0.03	1.827 ± 0.023	211.39	18.09
12:88 USAC	12	88	241.2 ± 1.7	11.15 ± 0.03	2.132 ± 0.022	208.89	21.33
23:77 USAC	23	77	246.9 ± 0.2	11.25 ± 0.03	2.114 ± 0.019	210.81	18.52
34:66 USAC	34	66	241.8 ± 2.7	11.21 ± 0.02	2.109 ± 0.028	208.24	17.86
47:53 USAC	47	53	248.1 ± 3.0	11.19 ± 0.04	2.008 ± 0.009	197.33	9.18
				Lenght (mm)	Section (mm^2^)		
45:55 HME	45	55	242.5 ± 6.1	47.22 ± 1.18	4.16 ± 0.00	196.48	10.92

**Table 2:** Normalized results of rheological parameters for PBS_CL powder with the mean incidence values.

Incidence Factor	Parameter	Symbol	Unit	Normalized Result	Mean Incidence
**Dimension**	Bulk density	ρbulk	g/mL	4.19	4.70
	Tapped density	ρtapped	g/mL	5.20	
**Compressibility**	Inter-particle Porosity	IP	-	3.83	3.85
	Carr Index	C	%	3.86	
**Flowability/** **powder** **flow**	Hausner Ratio	HR	-	2.42	4.37
	Angle of Repose	α	º	2.88	
	Powder flow	t”	s	7.80	
**Lubricity/** **stability**	Loss on Drying	%LOD	%	5.50	5.50
**Lubricity/** **dosage**	Particles ≤45 μm	Pf	%	9.98	6.50
	Homogeneity Index	Iθ	-	3.25	
**Parametric Index (PI)**		**0.40**			
**Parametric Profile Index (PPI)**		**4.89**			
**Good Compression Index (GCI)**		**5.29**			

**Table 3:** Drug release kinetic parameters and Excipient Efficiency from PBS_CL tablets and filaments. B = Higuchi kinetic constant; n = release exponent; kd = Peppas diffusion kinetic constant; kr = Peppas relaxation kinetic constant; r2 = determination coefficient.

Batch	Zero Order		Higuchi		Korsmeyer		Peppas y Sahlin			EE
	k0	r2	b (min^−1^)	r2	n	r2	kd (min^−5^)	kr (min^−1^)	r2	(min^1/2^)
12:88 DC	0.0083	0.9945	0.0844	0.9911	0.7065	0.9996	0.0844	0.1086	0.9911	12.23
23:77 DC	0.0056	0.9982	0.0563	0.9832	0.6277	0.9941	0.0563	0.0596	0.9832	
34:66 DC	0.0041	0.9992	0.0538	0.9742	0.5724	0.9724	0.0538	0.0637	0.9742	
47:53 DC	0.0042	0.986	0.0566	0.9951	0.5672	0.9973	0.0566	0.0451	0.9951	
12:88 USAC	0.0062	0.9894	0.0730	0.9477	0.8393	0.987	0.0758	0.1695	0.9682	25.47
23:77 USAC	0.0016	0.8902	0.0330	0.9867	0.5699	0.971	0.039	0.0112	0.9859	
34:66 USAC	0.0009	0.9468	0.0202	0.9885	0.4812	0.9921	0.0204	0.0041	0.9889	
47:53 USAC	0.0007	0.9432	0.0185	0.9985	0.4116	0.9983	0.0185	0.0313	0.9985	
45:55 HME	0.0008	0.9783	0.0167	0.9848	0.4886	0.9948	0.0205	−0.0001	0.9904	27.43

## Data Availability

Data is contained within the article.

## Supplementary Materials

The following are available online at https://www.mdpi.com/article/10.3390/pharmaceutics13071057/s1, Figure S1: Theophylline distribution of ionized/non ionized form, Figure S2: FTIR spectrum of PBS_CL copolyester with main absorption bands assigned to stretching (υ) and bending (δ) modes of different functional groups, Figure S3: ^13^C NMR spectrum of PBS_CL copolyester with peak assignments and expanded signals used for the determination of the degree of randomness, Figure S4: DSC thermograms of PBS_CL copolyester, Figure S5: High resolution SEM image of PBS_CL tablets obtained by USAC after drug release Detail of the nanostructured matrix at 1000×, Figure S6: High resolution SEM image of PBS_CL tablets obtained by USAC after drug release Detail of the nanostructured matrix at 5000×, Table S1: Molecular weights of PBS_CL copolyester, Table S2: Composition and microstructure of PBS_CL copolyester, Table S3: TGA parameters of PBS_CL copolyester, Table S4: Thermal properties of PBS_CL copolyester after the first heating run.
