# Supplementary material for: A Biodegradable Copolyester, Poly(butylene succinate-co-ε-caprolactone), as a High Efficiency Matrix Former for Controlled Release of Drugs
Source: Pharmaceutics. 2021 Jul 10;13(7):1057. doi: 10.3390/pharmaceutics13071057 (PMC8309084; doi:10.3390/pharmaceutics13071057)
Supplement: Supplementary file 1 [file pharmaceutics-13-01057-s001.zip › pharmaceutics-1271777 sup for conv.pdf]

pharmaceutics

Supplementary Materials: A Biodegradable Copolyester, Poly(Butylene Succinate-*co*-ε-Caprolactone), as High Efficiency Matrix Former for Controlled Release of Drugs

Eduardo Galdón, Mónica Millán-Jiménez, Gloria Mora-Castaño, Antxon Martínez de Ilarduya and Isidoro Caraballo

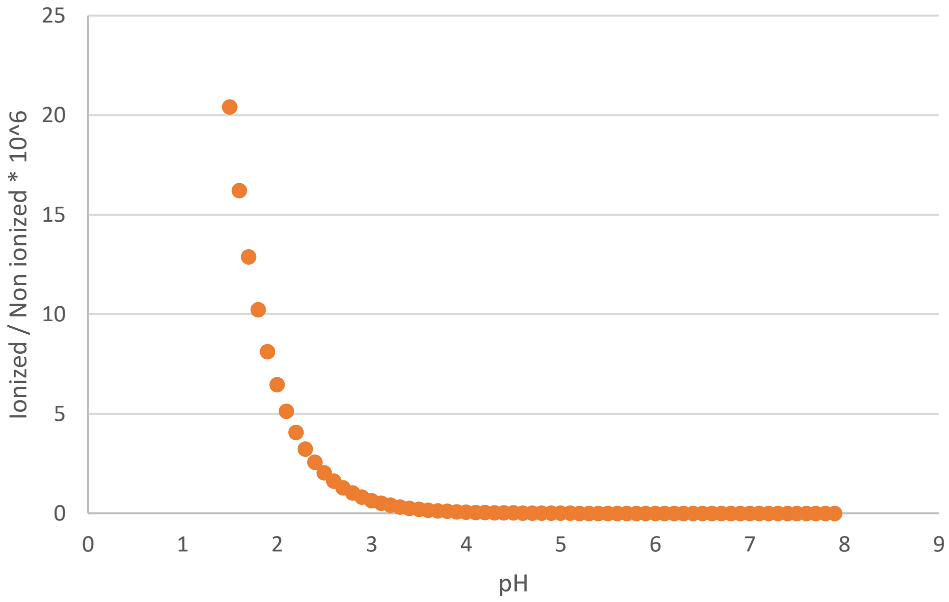

Figure S1. Theophylline distribution of ionized/non ionized form.

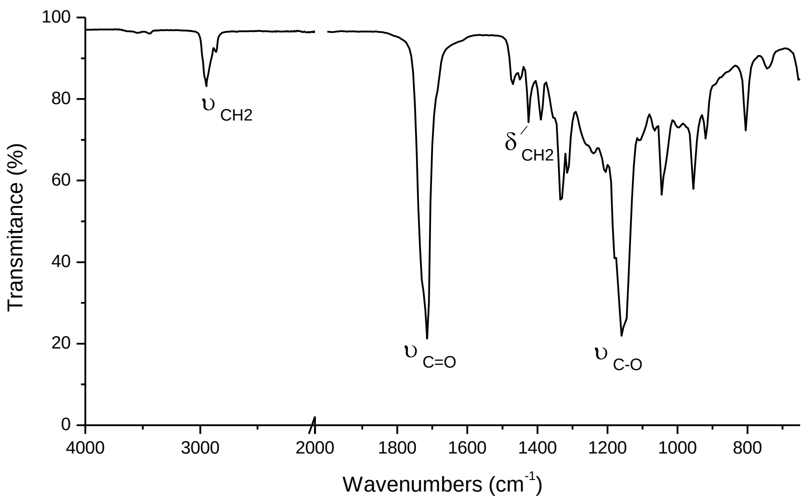

Figure S2. FTIR spectrum of PBS_CL copolyester with main absorption bands assigned to stretching (υ) and bending (δ) modes of different functional groups.

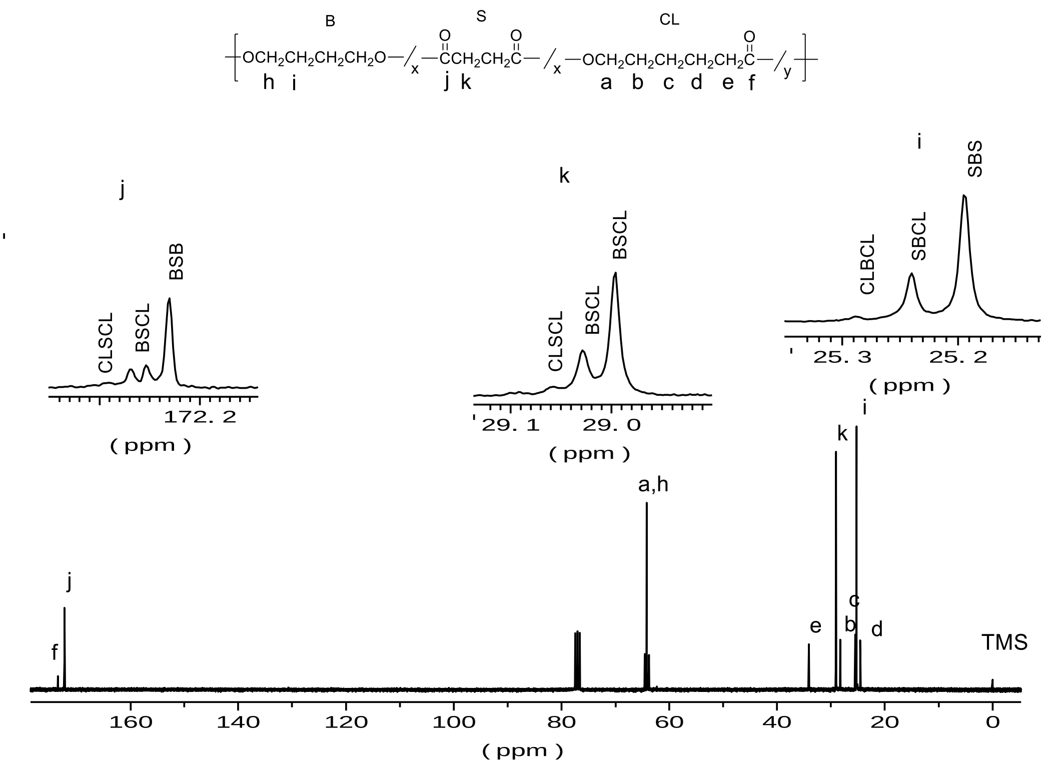

Figure S3. ^{13}C NMR spectrum of PBS_CL copolyester with peak assignments and expanded signals used for the determination of the degree of randomness.

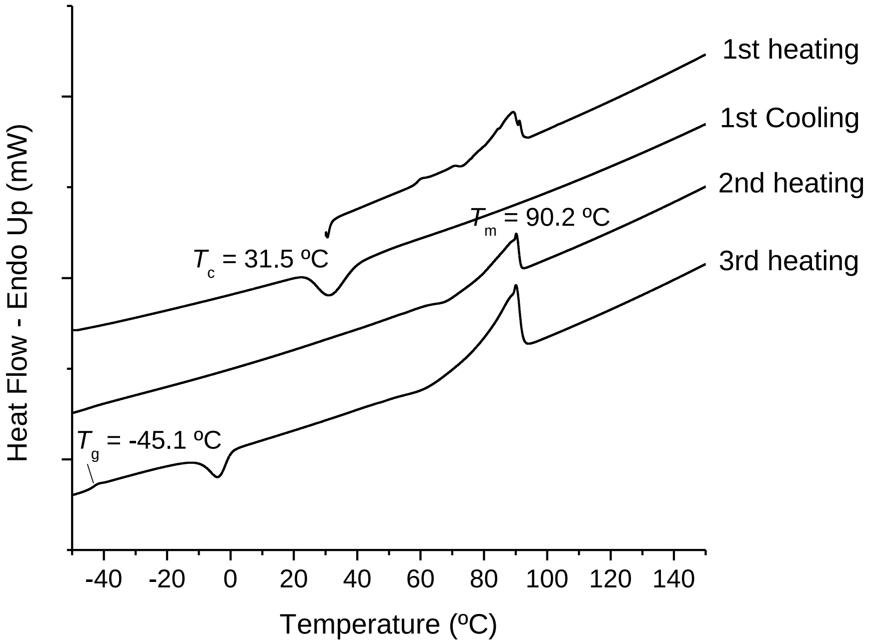

Figure S4. DSC thermograms of PBS_CL copolyester.

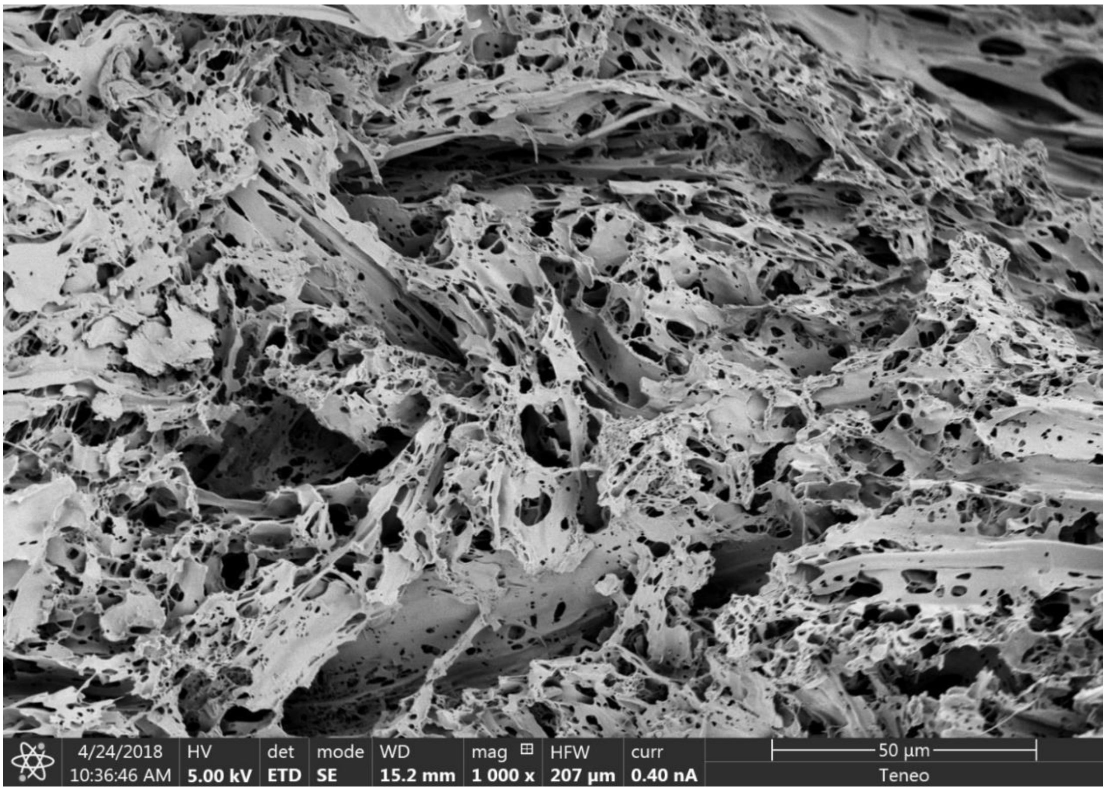

Figure S5. High resolution SEM image of PBS_CL tablets obtained by USAC (23:77 % v/v) after drug release. Detail of the nanostructured matrix at 1000x.

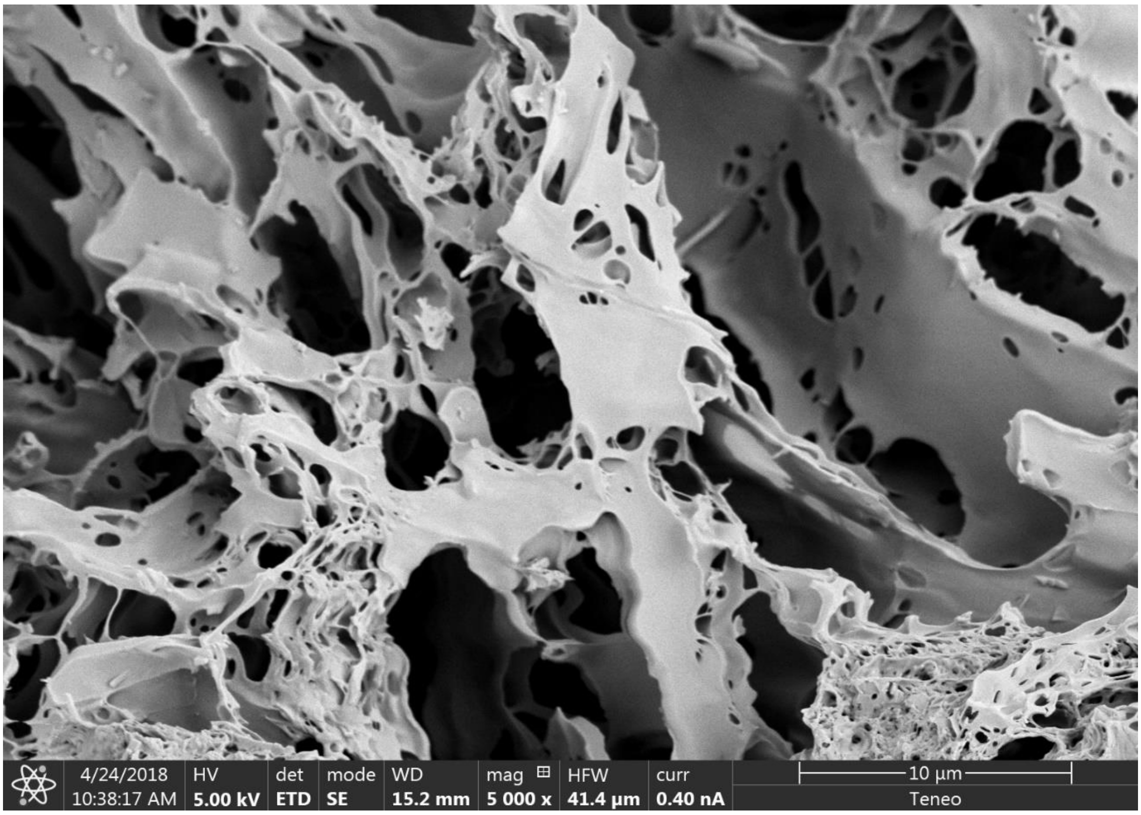

Figure S6. High resolution SEM image of PBS_CL tablets obtained by USAC (23:77 % v/v) after drug release. Detail of the nanostructured matrix at 5000x.

Table S1. Molecular weights of PBS_CL copolyester.

Copolymer	NMR	GPC		
	M_n (g/mol)	M_n (g/mol)	M_w (g/mol)	Đ
PBS_CL	21500	24300	51400	2.1

Table S2. Composition and microstructure of PBS_CL copolyester.

Copolymer	Composition (BS/CL mol/mol)		Microstructure (B-centered triad content)			
	Feed	Polymer	CLBCL	CLBS	SBS	R
PBS_CL	70/30	73,2/26,8	2.8	27.7	69.7	1.07

Table S3. TGA parameters of PBS_CL copolyester.

Copolymer	${}^{o}T_d^{10\%}$ (ºC)	${}^{max}T_d$ (ºC)	R_w (%)
PBS_CL	360	403	1,3

Table S4. Thermal properties of PBS_CL copolyester after the first heating run.

Copolymer	T_g (°C)	T_c (°C)	T_m (°C)	T_{cc} (°C)	ΔH_m (J/g)
PBS_CL	−45.1	31.5	90.2	-3.9	51.5
